# Research on Design of Tri-color Shift Device

**DOI:** 10.1186/s11671-016-1699-8

**Published:** 2016-11-03

**Authors:** Ping Xu, Xia Yuan, Haixuan Huang, Tuo Yang, Yanyan Huang, Tengfei Zhu, Shaotuo Tang, Wenda Peng

**Affiliations:** 1 College of Electronic Science and Technology, Shenzhen University, Shenzhen, 518060 China; 2 Institute of Micro-Nano Optoelectronic Technology, Shenzhen University, Shenzhen, 518060 China; 3 College of Optoelectronic Engineering, Shenzhen University, Shenzhen, 518060 China

**Keywords:** Subwavelength, Rectangular single periodic structure, Tri-color shift device, Anti-counterfeiting

## Abstract

An azimuth-tuned tri-color shift device based on an embedded subwavelength one-dimensional rectangular structure with single period is proposed. High reflection efficiencies for both TE and TM polarizations can be achieved simultaneously. Under an oblique incidence of 60°, the reflection efficiencies can reach up to 85, 86, and 100 % in blue (azimuth of 24°), green (azimuth of 63°), and red (azimuth of 90°) waveband, respectively. Furthermore, the laws of influence of device period, groove depth, coating thickness, and incident angle on reflection characteristics are investigated and exposed, and feasibility of the device is demonstrated. The proposed device realizes tri-color shift for natural light using a simple structure. It exhibits high efficiency as well as good security. Such a device can be fabricated by the existing embossing and coating technique. All these break through the limit of bi-color shift anti-counterfeiting technology and have great applications in the field of optically variable image security.

## Background

Based on the guided-mode resonance (GMR) theory, subwavelength gratings with feature sizes smaller than the working wavelength of incident light show some unique filtering performance, such as high efficiency, low sideband, tuneable resonance wavelength, and controllable line width. Such gratings can be applied as GMR filters [1–3], which are commonly fabricated by the modern technique of nano-manufacturing. Usually, the performance of the filter depends on the structure parameters and incident conditions; thus, visible wavelengths can be selectively reflected by changing the grating orientation, period, groove depth, and incident angle. This reflection results in an anisotropic color changing. In 1990, Gale et al. putted forward the authentication application of bi-color changing for the first time [4]. This technique was then industrialized by French Hologram Industries in 2003 and applied in management certificates for Ukrainian Ministry of Agriculture. To date, the bi-color shifting subwavelength gratings have found extensive and effective applications in the security areas including banknotes, passports, identity cards, and driving licenses [5–8]. However, with the popularity of bi-color shifting technology, its anti-counterfeiting function has declined. Therefore, how to further enhance the value of anti-counterfeiting and appreciation has attracted the attention of researchers in this field.

In recent years, various tri-color filters based on one-dimensional (1D) GMR gratings have been investigated and realized. Uddin et al. reported a multi-periodic color filter, in which three different grating periods generate respectively the red, green, and blue colors for reflected TE-polarized light [9]. Then, the multi-periodic grating turned by polarization state was presented [10]. With one period, the filter reflects green (TE) and blue (TM); while with the other one, the filter generates red (TE) and yellow (TM). Besides one-layer structure gratings, a multi-layered subwavelength grating was also proposed [11], where the reflective characteristics of the TE-polarized light, TM-polarized light, and white light are red, green, and yellow, respectively. Pesala and Madhusudan presented an asymmetric high contrast subwavelength grating (HCG) with two ridges [12]. For the 0th diffraction, red light is achieved at the normal viewing angle; while for the first-order diffractions, the respective output lights are blue and green at the left and right glancing angles. However, the mentioned devices based on multi-period, multi-layer, or asymmetric structures are difficult to be fabricated and have a high manufacturing cost. In order to simplify the structures, Uddin et al. reported a single periodic tri-color filter, which was obtained by tuning the incident angles of TE polarization [13].

Although the tri-color filters mentioned above exhibit an increased reflected color, they are inevitably susceptible to polarization dependency. That means only TE or TM polarization should be reflected highly in specified wave band, which will loss 50 % energy of input light. Meanwhile, polarizers have difficulty to satisfy the popular anti-counterfeiting technology with the naked eye. Usually, 2D grating structures are used to realize the polarization-independent filtering [14–17], but they have the relative complexity of production process. Besides, the naked structure of the above tri-color filters can be easily replicated. All these reasons cause an obstacle to expand the application of the existing tri-color filters in the security area.

Therefore, it is very significant to study an azimuth-tuned tri-color shift device with 1D single period simple structure, which can simultaneously realize high reflection efficiencies for both TE and TM polarizations in specified bands. What is more, the device not only has anti-counterfeiting function but also can be easily fabricated. However, we have not seen the relevant report at present.

Our groups have done a lot of researches on binary optics in recent years [18–23]. Here, we report a tri-color shift device by using embedded subwavelength 1D rectangular structure with single period. Rigorous coupled wave analysis (RCWA) and genetic algorithm (GA) are used for designing. The proposed device could work well on a natural input light. By tuning the azimuths, the device exhibits a tri-color shift of blue, green, and red for both TE and TM polarizations simultaneously. Moreover, high reflectivities of 85, 86, and 100 % for blue, green, and red color can be reached, respectively. The proposed device realizes tri-color shift for natural light using a simple structure, which breaks through the limit of the bi-color shift anti-counterfeiting technology. Mature embossing and coating technologies suggest a promising prospect in the application of optically variable image security.

## Methods

**Fig. 1:**
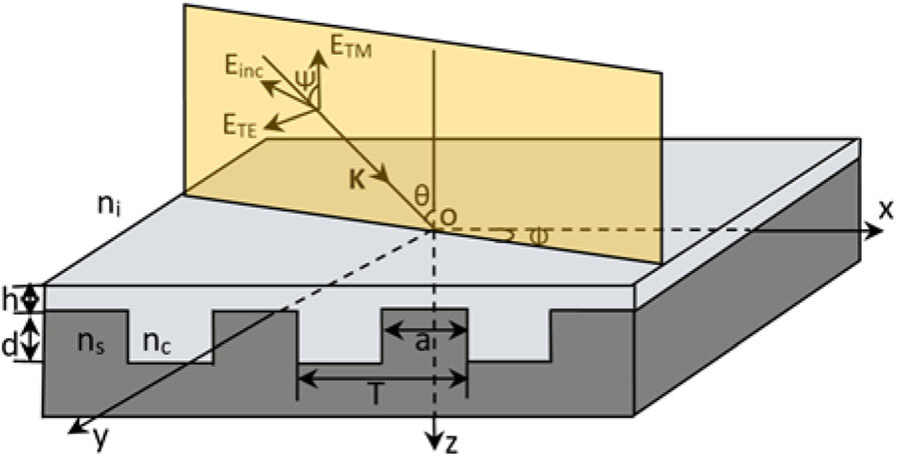
Structure model of the tri-color shift device

The structure model of the tri-color shift device is shown in Fig. 1. *T* is the grating period, *a* is the ridge width, *d* is the groove depth, and *h* refers to the thickness of the coating. The coating which covers on the top of an authentication device is used to avoid coping and has the effect of raising the reflection efficiency. Here, the grating period is shorter than the optical wavelength and the duty ratio *f* is defined as *f* = *a/T*. As shown in Fig. 1, the normal to the boundary is in the *z* direction, and the grating vector is in the *x* direction. Along the *z* axis, the region of the grating can be divided into four layers: the region I (*z < -h*) is the incident medium layer with refractive index *n*_*i*_, the region II (*-h ≤ z <0*) is the coating layer with refractive index *n*_*c*_, the region III (*0 ≤ z ≤ d*) is the rectangular grating layer with refractive indices *n*_*c*_ and *n*_*s*_, and the region IV (*z > d*) is the substrate layer with refractive index *n*_*s*_. A polarized plane wave with wavelength *λ* (*λ > T*) and wave vector *k* is incident upon the subwavelength structure. The flat composed by wave vector *k* and *z* axis is the incident plane. Azimuth angle *ϕ*, incident angle *θ*, and polarized angle *ψ* are angles between the incident plane and plane *xoz*, wave vector *k* and *z* axis, polarized direction of incident light and incident plane, respectively*.* The component of the electric vector vertical to the incident plane is TE polarization and parallel to it is TM polarization.

Since the scalar diffraction theory is no longer suitable in subwavelength scale, RCWA, a vector analysis method suitable for the periodic subwavelength structure, is utilized to analyze the diffraction of the tri-color shift device [24]. Firstly, the parsing expressions of electromagnetic field in the incident medium, the coating medium, and the substrate medium are derived from the Maxwell equations; Subsequently, by operating Fourier expanding to the dielectric constant and electromagnetic field of the grating region, and applying the boundary conditions of electromagnetic fields for different boundaries, a series of infinite dimension couple-wave differential equations are obtained; Finally, eigenvalue methods are used to solve the equations, and numerical solutions to the diffraction efficiency of each diffractive wave, *η*_*m*_, are figured out [25]. The above-mentioned structure is in the scale of subwavelength, *λ > T*; therefore, the grating will only exist in the 0th diffraction light [26].

As the natural light can be decomposed into two polarizations with their vibration directions vertical, light intensities equal, and phase relationships unfixed, it can be considered as a superposition of vertical TE and parallel TM polarization relative to the incident plane [6, 27]. To achieve the azimuth-tuned tri-color shift of blue, green, and red for natural input light, waveband and magnitude of the reflection peaks for TE and TM polarizations should be designed at three azimuths. That is, high reflectivity should be reached in blue band at the first azimuth, in green band at the second azimuth, and in red band at the third azimuth for both TE and TM polarizations.

Numerical solution to the 0th reflection efficiency is relative to the incident light parameters (*λ*, *ψ*, *ϕ*, *θ*), the structure parameters of the device (*f*, *T*, *d*, *h*), as well as the refractive indices of all the regions (*n*_*i*_, *n*_*c*_, *n*_*s*_). There is no analytical relationship between these parameters and the 0th reflection efficiency. And this makes it very difficult to design the device—by varying the parameters step-by-step to achieve the designing goal is almost impossible. With the mechanism of jumping out of local extreme point, GA is especially suitable for solving multi-variable and discrete variable optimization problems [28, 29]. In this paper, the key parameters influencing on reflection efficiency of the tri-color shift device are optimized by combining RCWA and GA.

To reduce the variables, we fix some parameters such as *θ*, *f*, *n*_*i*_, *n*_*c*_, *n*_*s*_. Thus, the key design procedure of the tri-color shift device is to optimize *ϕ*, *T*, *d*, and *h* to reach the high reflection efficiencies in blue, green, and red bands for both TE and TM polarizations, respectively. Considering the design goals above, the evaluation function can be established as follows:

1 $$ \mathrm{M}\mathrm{F}\kern0.5em \left({\phi}_b,{\phi}_g,{\phi}_r,\kern0.5em d,\kern0.5em T,\kern0.5em h\right)\kern0.5em =\kern0.5em {\mathrm{MF}}_{\_b}\kern0.5em +\kern0.5em {\mathrm{MF}}_{\_g}\kern0.5em +\kern0.5em {\mathrm{MF}}_{\_r} $$

where, *ϕ*_*b*_, *ϕ*_*g*_, *ϕ*_*r*_ are azimuth angles where approaching the high reflectivity in blue, green, and red bands, respectively, and MF*_*_*b*_, MF*_*_*g*_, MF*_*_*r*_ are sub-functions of evaluation function *MF*. The definition of each sub-evaluation function is the key of the optimization. In order to show respectively blue, green, and red colors at *ϕ*_*b*_, *ϕ*_*g*_, *ϕ*_*r*_, high reflectivity in one band and low reflectivities in other two bands should be realized. According to this principle, the evaluation function can be set as follows:

2 $$ \left\{\begin{array}{l}\mathrm{M}\mathrm{F}{\_}_b=\alpha \times {\displaystyle \sum_{i=1}^{L_1}{\left[1-\eta \left({\lambda}_i,0{}^{\circ},{\phi}_b,d,T,h\right)\right]}^2}+\beta \times {\displaystyle \sum_{j=1}^{L_2}{\left[0-\eta \left({\lambda}_j,0{}^{\circ},{\phi}_g,d,T,h\right)\right]}^2}+\gamma \times {\displaystyle \sum_{k=1}^{L_3}{\left[0-\eta \left({\lambda}_k,0{}^{\circ},{\phi}_r,d,T,h\right)\right]}^2}+{\alpha}^{\hbox{'}}\times {\displaystyle \sum_{i=1}^{L_1}{\left[1-\eta \left({\lambda}_i,90{}^{\circ},{\phi}_b,d,T,h\right)\right]}^2}\\ {}+{\beta}^{\hbox{'}}\times {\displaystyle \sum_{j=1}^{L_2}{\left[0-\eta \left({\lambda}_j,90{}^{\circ},{\phi}_g,d,T,h\right)\right]}^2}+{\gamma}^{\hbox{'}}\times {\displaystyle \sum_{k=1}^{L_3}{\left[0-\eta \left({\lambda}_k,90{}^{\circ},{\phi}_r,d,T,h\right)\right]}^2}\\ {}\mathrm{M}\mathrm{F}{\_}_g=\alpha \times {\displaystyle \sum_{j=1}^{L_2}{\left[1-\eta \left({\lambda}_j,0{}^{\circ},{\phi}_g,d,T,h\right)\right]}^2}+\beta \times {\displaystyle \sum_{i=1}^{L_1}{\left[0-\eta \left({\lambda}_i,0{}^{\circ},{\phi}_b,d,T,h\right)\right]}^2}+\gamma \times {\displaystyle \sum_{k=1}^{L_3}{\left[0-\eta \left({\lambda}_k,0{}^{\circ},{\phi}_r,d,T,h\right)\right]}^2}+{\alpha}^{\hbox{'}}\times {\displaystyle \sum_{j=1}^{L_2}{\left[1-\eta \left({\lambda}_j,90{}^{\circ},{\phi}_g,d,T,h\right)\right]}^2}\\ {}+{\beta}^{\hbox{'}}\times {\displaystyle \sum_{i=1}^{L_1}{\left[0-\eta \left({\lambda}_i,90{}^{\circ},{\phi}_b,d,T,h\right)\right]}^2}+{\gamma}^{\hbox{'}}\times {\displaystyle \sum_{k=1}^{L_3}{\left[0-\eta \left({\lambda}_k,90{}^{\circ},{\phi}_r,d,T,h\right)\right]}^2}\\ {}\mathrm{M}\mathrm{F}{\_}_r=\alpha \times {\displaystyle \sum_{k=1}^{L_3}{\left[1-\eta \left({\lambda}_k,0{}^{\circ},{\phi}_r,d,T,h\right)\right]}^2}+\beta \times {\displaystyle \sum_{j=1}^{L_2}{\left[0-\eta \left({\lambda}_j,0{}^{\circ},{\phi}_g,d,T,h\right)\right]}^2}+\gamma \times {\displaystyle \sum_{i=1}^{L_1}{\left[0-\eta \left({\lambda}_i,0{}^{\circ},{\phi}_b,d,T,h\right)\right]}^2}+{\alpha}^{\hbox{'}}\times {\displaystyle \sum_{k=1}^{L_3}{\left[1-\eta \left({\lambda}_k,90{}^{\circ},{\phi}_r,d,T,h\right)\right]}^2}\\ {}+{\beta}^{\hbox{'}}\times {\displaystyle \sum_{j=1}^{L_2}{\left[0-\eta \left({\lambda}_j,90{}^{\circ},{\phi}_g,d,T,h\right)\right]}^2}+{\gamma}^{\hbox{'}}\times {\displaystyle \sum_{i=1}^{L_1}{\left[0-\eta \left({\lambda}_i,90{}^{\circ},{\phi}_b,d,T,h\right)\right]}^2}\end{array}\right. $$

where 0°, 90° are polarized angles of TM and TE polarization, respectively; *L*_1_, *L*_2_, *L*_3_ are numbers of wavelength samplings in blue, green, and red bands, respectively; *λ*_*i*_, *λ*_*j*_, *λ*_*k*_ are the wavelength samplings in above three bands (*i* = 1,2,…,L1, *j* = 1,2,…,L2, *k* = 1,2,…,L3), respectively; and *α*, *β*, *γ*, *α’*, *β’*, *γ’* are the weight factors ranging from 0 to 1 and set according to design demand, whose sum is 1. In sub-functions MF*_*_*b*_, the first item denotes the difference between the maximum reflectivity and the real reflectivity of TM polarization in blue band; the second and third items denote the differences between the real reflectivity and the minimum reflectivity of TM polarization in green and red bands; the forth item denotes the difference between the maximum reflectivity and the real reflectivity of TE polarization in blue band; the last two items denote the differences between the real reflectivity and the minimum reflectivity of TE polarization in green and red bands. The definitions of MF*_*_*g*_ and MF*_*_*r*_ are analogous. Therefore, the focus is on searching for a set of optimal parameters *ϕ*_*b*_, *ϕ*_*g*_, *ϕ*_*r*_, *d*, *T*, *h* to get a minimum evaluated function MF.

First, the initial population is produced by setting the initial values of *ϕ*_*b*_, *ϕ*_*g*_, *ϕ*_*r*_, *d*, *T*, *h*. Next, whether the convergence conditions are satisfied should be judged by calculating the 0th reflection efficiency as well as the evaluation function MF. If the convergence conditions are not satisfied, selection, intersection, and mutation are done to produce the new population. Then, the evaluation function MF is recalculated and the convergence conditions are rejudged. Finally, the optimized parameters of *ϕ*_*b*_, *ϕ*_*g*_, *ϕ*_*r*_, *d*, *T*, *h* are output until the convergence conditions are satisfied. The optimal structure parameters of the device and the azimuths of incident light then can be obtained.

## Results

With the optimization methods above, a tri-color shift device using an embedded subwavelength simple structure is designed to realize azimuth-tuned color shift of blue, green, and red lights. For convenient fabrication, we set the duty ratio *f* = 0.5 and the incident medium is air with *n*_*i*_ = 1.0. Incident angle and the refractive index of materials are also important parameters but not our research focus. So they are set as below. The incident angle *θ* = 60°, the substrate is polyethylene terephthalate (PET) polyester with *n*_*s*_ = 1.65, and the overlay is ZnO with *n*_*c*_ = 2.0. With many times of iterative optimization, the optimal parameters which satisfy all the design requirements are obtained. *T =* 470.8 nm, *d =* 153.1 nm, *h =* 83.3 nm with *ϕ*_*b*_ = 24°, *ϕ*_*g*_ = 63°, and *ϕ*_*r*_ = 90°. It is the result of special parameters that TE and TM polarizations achieve high reflection efficiencies simultaneously in three specified wave bands by such a simple structure.

**Fig. 2:**
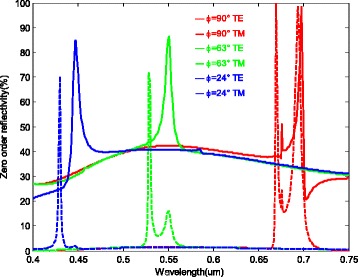
Reflectivity spectra of the tri-color shift device for TE and TM polarizations at three azimuths (*θ* = 60°, *T =* 470.8 nm, *d =* 153.1 nm, *h =* 83.3 nm, *f* = 0.5)

When TE and TM polarizations are incident at *θ* = 60°, their simultaneous reflectance spectra in visible wavelengths [400 nm, 750 nm] at three azimuths are shown in Fig. 2. As can be seen from Fig. 2, when *ϕ*_*b*_ = 24°, TE and TM polarizations have reflection peaks at 447 and 430 nm, respectively. Although the TE and TM peaks are 17 nm apart, they are all in blue band. So the device will reflect blue light. When *ϕ*_*g*_ = 63°, TE and TM polarizations have reflection peaks at 551 and 529 nm, respectively. The gap between TE and TM peaks is 21 nm. In a similar way, the device will still reflect green light. When *ϕ*_*r*_ = 90°, TE and TM polarizations have reflection peaks at 676 and 698 nm and 670 and 694 nm, respectively. And the device will reflect red light. The peak reflectivities are denoted in Table 1, with the highest being up to 85, 86, and 100 % in blue (*ϕ*_*b*_ = 24°), green (*ϕ*_*g*_ = 63°), and red (*ϕ*_*r*_ = 90°) bands, respectively.

**Table 1:** The peak reflectivities of blue (*ϕ*_*b*_ *=* 24°), green (*ϕ*_*g*_ *=* 63°), and red (*ϕ*_*r*_ *=* 90°) lights

Azimuth (degrees)	Wavelength band (nm)	Reflectivity of TE polarization (%)	Reflectivity of TM polarization (%)
24	430~450	85	70
63	520~560	86	72
90	670~700	51, 99	100, 99

As a result, when the nature light is incident with angle of 60°, the device exhibits blue, green, and red color responses at azimuth of 24°, 63°, and 90°, respectively. In spite of the tiny displacements of resonance peaks caused by dispersion of the material, visual effects of the device are not affected, since the proposed filter is an optical variable device perceived by human vision instead of a narrow band filter. In addition, the absorption of the material can be ignored because of the high reflectivity.

## Discussion

What color the tri-color shift device will display as well as what diffraction efficiency it has depends on the parameters of the incident light and the device structure. In any process of manufacture and observation, there are some deviations in device structure and incident angle, which means variation of the boundary condition of electromagnetic field. This will lead to variation of the numerical solution to the 0th reflection efficiency, that is, the reflection peak, and affected the anti-counterfeiting function of the tri-color shift device. Therefore, it is necessary to analyze the effects of the changing of device period, groove depth, coating thickness, and incident angle on the color and the reflectivity of the device and explore the laws of influence of key parameters on the design results, which plays an important role in designing, manufacturing and testing of the device.

### Influence of Device Period on the Reflection Characteristics

**Fig. 3:**
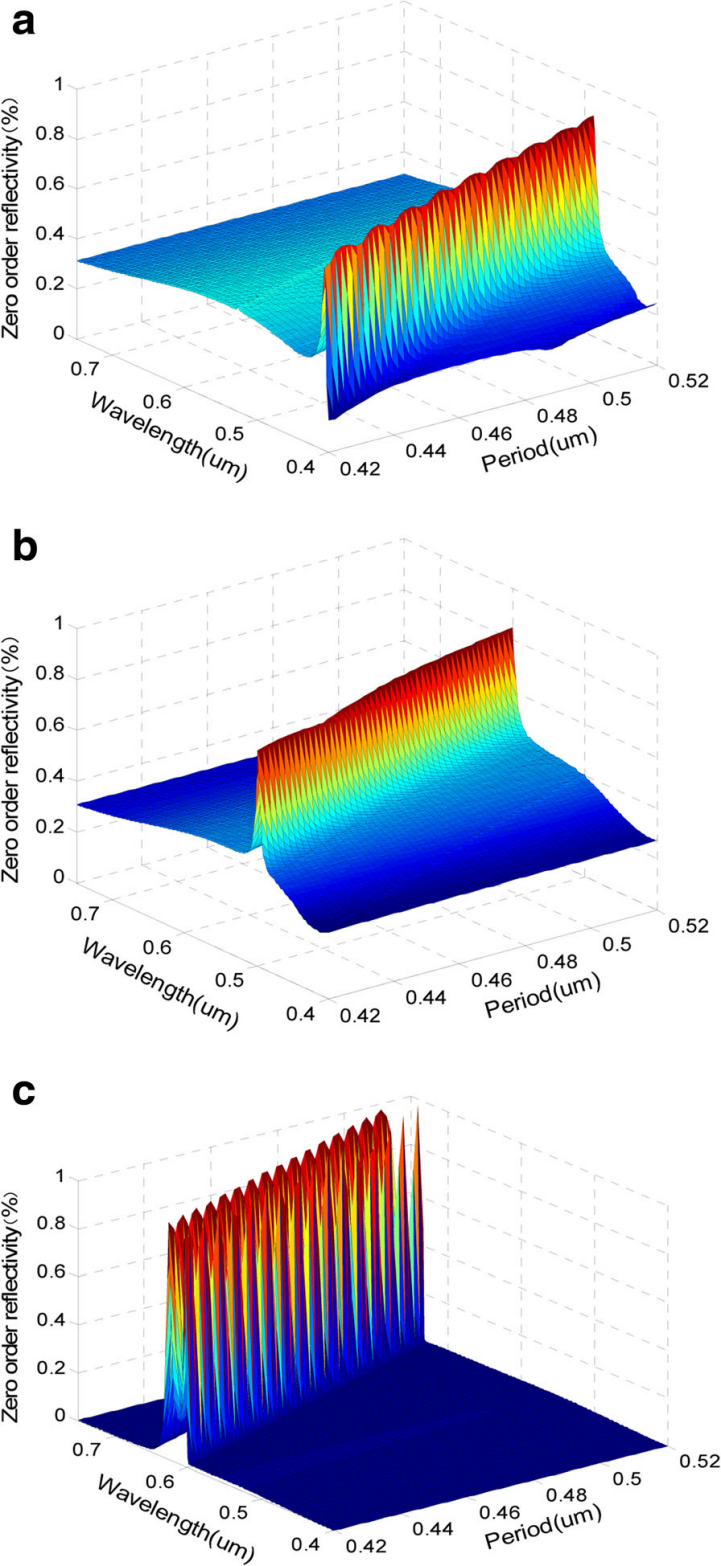
The change of reflection peaks with the device period. **a** *∅*_*b*_ = 24°. **b** *∅*_*g*_ = 63°. **c** *∅*_*r*_ = 90°. (*θ* = 60°, *T* = 470.8 nm, *d* = 153.1 nm, *h* = 83.3 nm, *f* = 0.5)

Figure 3 and Table 2 show the effects of the period deviation on the reflection peaks of the tri-color shift device. When other parameters are fixed to design values, with the period increases, the peak reflectivities will shift toward long wavelengths. When the period shifts by ±5 %, i.e., ± 23 nm relative to the design value, the peak reflectivities of blue, green, and red lights still can be obtained respectively in the ranges of [428 nm, 466 nm], [528 nm, 574 nm], and [664 nm, 724 nm] in the direction of the original three azimuths.

**Table 2:** The effects of period deviation on wavelength and value of the reflection peaks at three azimuths

Azimuth (degrees)	Period deviation (nm)	Wavelength (nm)	Reflectivity (%)	Color
24	−23	428	82	Blue
	+23	466	87	
63	−23	528	84	Green
	+23	574	86	
90	−23	664	98	Red
	+23	724	93	

### Influence of Groove Depth on the Reflection Characteristics

**Fig. 4:**
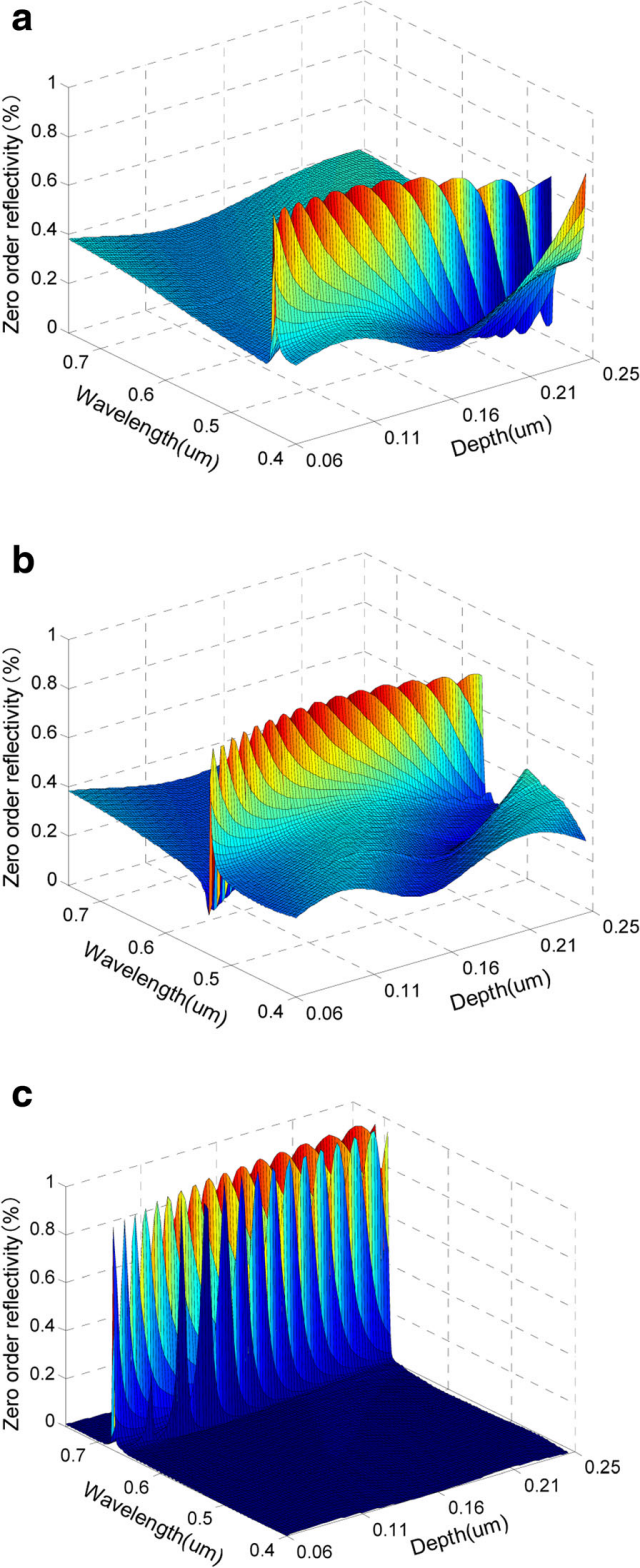
The change of reflection peaks with the groove depth. **a** *∅*_*b*_ = 24°. **b** *∅*_*g*_ = 63°. **c** *∅*_*r*_ = 90°. (*θ* = 60°, *T* = 470.8 nm, *d* = 153.1 nm, *h* = 83.3 nm, *f* = 0.5)

The changes of reflection peaks with the groove depth are given in Fig. 4 and Table 3. When other parameters are fixed to design values, with the groove depth increases, the peak reflectivities will shift toward long wavelengths. When the groove depth shifts by ±48 %, i.e., ±73 nm relative to the design value, the peak reflectivities of blue, green, and red lights still can be obtained respectively in the ranges of [438 nm, 454 nm], [536 nm, 560 nm], and [680 nm, 704 nm] in the direction of the original three azimuths.

**Table 3:** The effects of depth deviation on wavelength and value of the reflection peaks at three azimuths

Azimuth (degrees)	Period deviation (nm)	Wavelength (nm)	Reflectivity (%)	Color
24	−73	438	89	Blue
	+73	454	64	
63	−73	536	78	Green
	+73	560	78	
90	−73	680	90	Red
	+73	704	97	

### Influence of Coating Thickness on the Reflection Characteristics

**Fig. 5:**
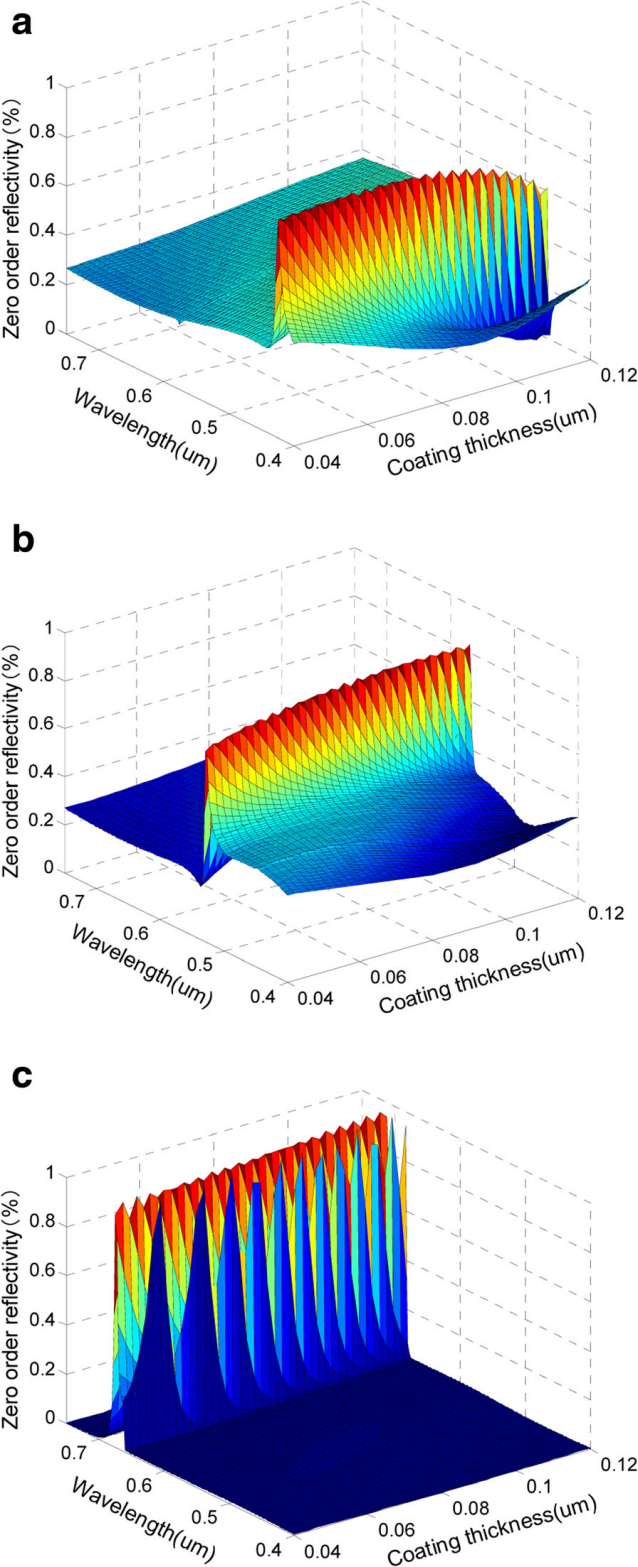
The change of reflection peaks with the coating thickness. **a** *∅*_*b*_ = 24°. **b** *∅*_*g*_ = 63°. **c** *∅*_*r*_ = 90°. (*θ* = 60°, *T* = 470.8 nm, *d* = 153.1 nm, *h* = 83.3 nm, *f* = 0.5)

Figure 5 and Table 4 reflect the dependences of the reflection peaks on coating thickness. When other parameters are fixed to the design values, with the thickness of the coating increases, the peak reflectivities will shift toward long wavelengths. When the thickness shifts by ±40 %, i.e., ±33 nm relative to the design value, the peak reflectivities of blue, green, and red lights can be obtained respectively in the ranges of [430 nm, 464 nm], [532 nm, 568 nm], and [678 nm, 710 nm] in the direction of the original three azimuths.

**Table 4:** The effects of thickness deviation on wavelength and value of the reflection peaks at three azimuths

Azimuth (degrees)	Period deviation (nm)	Wavelength (nm)	Reflectivity (%)	Color
24	−33	430	88	Blue
	+33	464	66	
63	−33	532	83	Green
	+33	568	84	
90	−33	678	98	Red
	+33	710	94	

### Influence of Incident Angle on the Reflection Characteristics

**Fig. 6:**
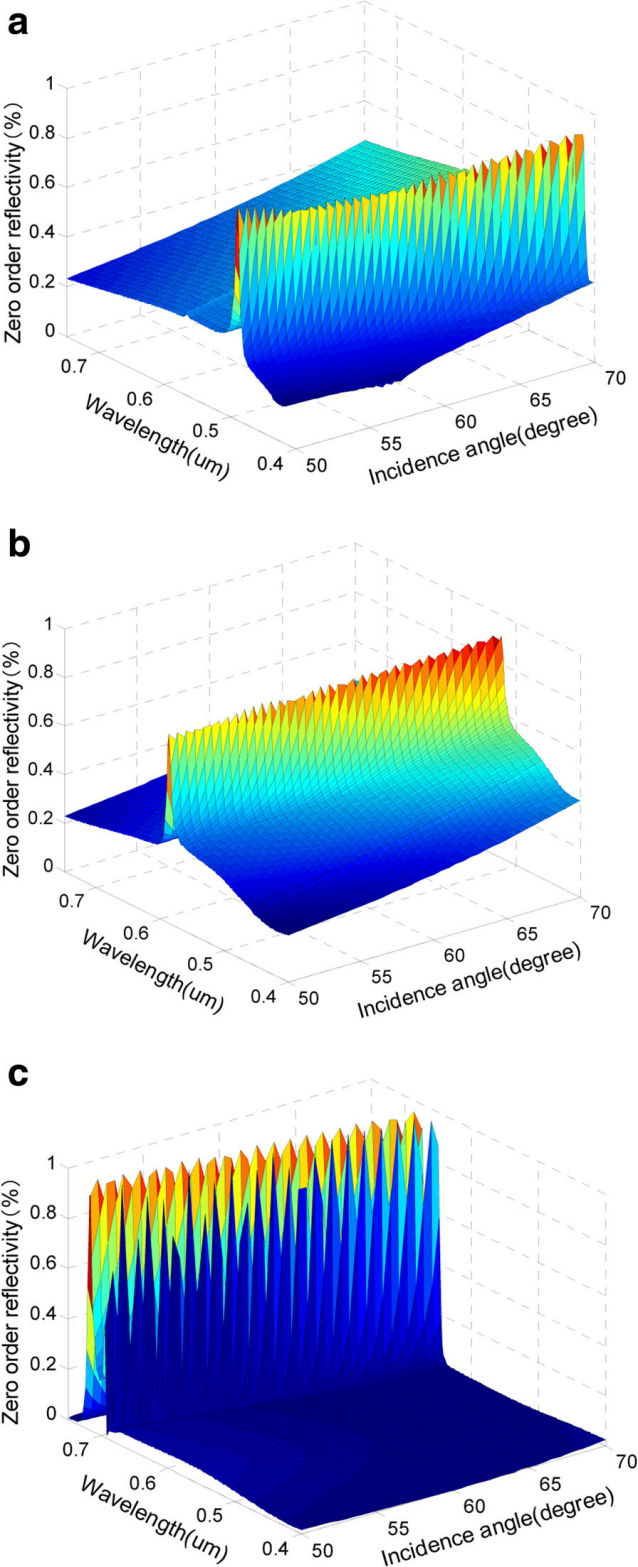
The change of reflection peaks with the incident angle. **a** *∅*_*b*_ = 24°. **b** *∅*_*g*_ = 63°. **c** *∅*_*r*_ = 90°. (*θ* = 60°, *T* = 470.8 nm, *d* = 153.1 nm, *h* = 83.3 nm, *f* = 0.5)

The dependences of reflection peaks on the incident angle are shown in Fig. 6 and Table 5. When other parameters are fixed to the original designs, with the incident angle increases, the peak reflectivities will shift toward short wavelengths. When the angle shifts by ±10 %, i.e., ±6° relative to the design value, the peak reflectivities of blue, green, and red lights can be obtained respectively in the ranges of [428 nm, 470 nm], [532 nm, 572 nm], and [682 nm, 708 nm] in the direction of the original three azimuths.

**Table 5:** The effects of incident angle deviation on wavelength and value of the reflection peaks at three azimuths

Azimuth (degrees)	Period deviation (nm)	Wavelength (nm)	Reflectivity (%)	Color
24	−6	470	82	Blue
	+6	428	89	
63	−6	572	82	Green
	+6	532	89	
90	−6	708	98	Red
	+6	682	98	

In a word, with the increase of device period, groove depth, coating thickness, and the decrease of incident angle, the reflection peaks of blue, green, and red lights get red shift. Changes of groove depth and coating thickness have little impact on peak. When the period, groove depth, coating thickness, and the incident angle varies by ±5, ±48, ±40, and ±10 %, i.e., ±23, ±73, and ±33 nm, and ±6° relative to the original designs, respectively, the device can well keep the color-shift effect of blue, green, and red. Therefore, to get a good anti-counterfeiting effect, the deviation of period must be strictly controlled during manufacturing. According to the current technology of electron beam lithography, the period precision can be satisfied completely. The groove depth and the coating thickness have high redundancy, so the common technologies of etching, embossing, and coating can be used to fabricate the tri-color shift device.

Since the resonance of subwavelength grating depends on its physical parameters, reflection peaks of the device shift with the change of above parameters. Due to space limitations, we will explain the physical mechanism in other article.

## Conclusions

In this paper, an azimuth-tuned tri-color shift device using an embedded subwavelength 1D rectangular structure with single period is proposed. When TE and TM polarizations are incident at angle of 60°, the blue, green, and red lights can achieve the highest reflectivity of 85 (*ϕ* = 24°), 86 (*ϕ* = 63°), 100 % (*ϕ* = 90°), respectively. By rotating the device in its plane, it yields a nontrivial and interesting variation of colors. Tolerance analysis shows that the device period, groove depth, coating thickness, and incident angle are allowed to vary respectively by ±5, ±48, ±40, and ±10 %, i.e., ± 23, ±73, and ±33 nm, and ±6° relative to the design values in fabrication. The results prove the producing feasibility and provide the guidance to design and manufacture of the device.

This tri-color shift device breaks through the limit of bi-color shifting technology. The device is hard to be replicated with special design principle, embedded structure, and high-precision manufacture, while its 1D single period structure is good for manufacture of the high-precision master mask. Moreover, the device can be mass produced at low cost by matured embossing technique. It generates high reflection efficiencies for TE and TM polarizations simultaneously. So the general public can conveniently observe the unique visual effects in daylight. Also, the trained examiners can readily identify the distinctive spectral behaviors by machine. At last, viewing the device is enjoyable because of the novel visual effect. So the tri-color shift device may have great applications in the field of the optically variable image security.
